# 10-Acetyl-10*H*-phenothia­zine 5-oxide

**DOI:** 10.1107/S1600536809028487

**Published:** 2009-07-25

**Authors:** Qiang Wang, Lei Yang, Zhouqing Xu, Yanchun Sun

**Affiliations:** a Department of Physics and Chemistry, Henan Polytechnic University, Jiao Zuo 454000, People’s Republic of China; b Department of Medicine, Hebi College of Vocation and Technology, He Bi 458030, People’s Republic of China

## Abstract

In the title compound, C_14_H_11_NO_2_S, the sulfoxide O atom is disordered over two sites with occupancies of 0.886 (4) and 0.114 (4), reflecting a partial inversion of the lone pair at the tetra­hedral S-atom site. In the crystal, a supra­molecular arrangement arises from weak inter­molecular C—H⋯O hydrogen bonds. π–π contacts between the aromatic rings of symmetry-related mol­ecules [centroid–centroid distances = 3.7547 (15) and 3.9577 (15) Å] in parallel accumulation further stabilize the crystal structure.

## Related literature

For synthetic details, see: Gilman & Nelson (1953 ▶); Chan *et al.* (1998 ▶). For a general background to phenothia­zine-based mol­ecules, see: Miller *et al.* (1999 ▶); Lam *et al.* (2001 ▶); Wermuth (2003 ▶); Wang *et al.* (2008 ▶).
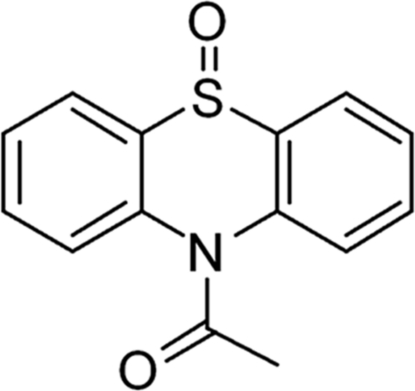


## Experimental

### 

#### Crystal data

C_14_H_11_NO_2_S
*M* *_r_* = 257.30
Monoclinic, 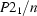

*a* = 8.1244 (1) Å
*b* = 14.1787 (2) Å
*c* = 10.7576 (1) Å
β = 100.963 (1)°
*V* = 1216.59 (3) Å^3^
*Z* = 4
Mo *K*α radiation
μ = 0.26 mm^−1^
*T* = 296 K
0.20 × 0.14 × 0.13 mm

#### Data collection

Bruker APEXII CCD area-detector diffractometer
Absorption correction: multi-scan (*SADABS*; Sheldrick, 2003 ▶) *T*_min_ = 0.950, *T*_max_ = 0.967
11538 measured reflections
3067 independent reflections
2404 reflections with *I* > 2σ(*I*)
*R*_int_ = 0.019

#### Refinement

*R*[*F* ^2^ > 2σ(*F* ^2^)] = 0.046
*wR*(*F* ^2^) = 0.127
*S* = 1.09
3067 reflections
174 parameters
2 restraints
H-atom parameters constrained
Δρ_max_ = 0.34 e Å^−3^
Δρ_min_ = −0.24 e Å^−3^

### 

Data collection: *APEX2* (Bruker, 2003 ▶); cell refinement: *SAINT* (Bruker, 2003 ▶); data reduction: *SAINT*; program(s) used to solve structure: *SHELXS97* (Sheldrick, 2008 ▶); program(s) used to refine structure: *SHELXL97* (Sheldrick, 2008 ▶); molecular graphics: *SHELXTL* (Sheldrick, 2008 ▶); software used to prepare material for publication: *SHELXTL*.

## Supplementary Material

Crystal structure: contains datablocks I, global. DOI: 10.1107/S1600536809028487/bh2235sup1.cif

Structure factors: contains datablocks I. DOI: 10.1107/S1600536809028487/bh2235Isup2.hkl

Additional supplementary materials:  crystallographic information; 3D view; checkCIF report

## Figures and Tables

**Table 1:** Hydrogen-bond geometry (Å, °)

*D*—H⋯*A*	*D*—H	H⋯*A*	*D*⋯*A*	*D*—H⋯*A*
C5—H5*A*⋯O1*A*^i^	0.93	2.31	3.207 (3)	163

Symmetry code: (i) 
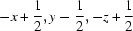
.

**Fig. 1.**
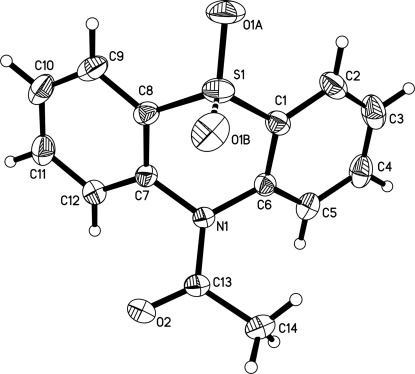
The molecular structure of the title molecule, with the atom-numbering scheme. Displacement ellipsoids are drawn at the 30% probability level.

**Fig. 2.**
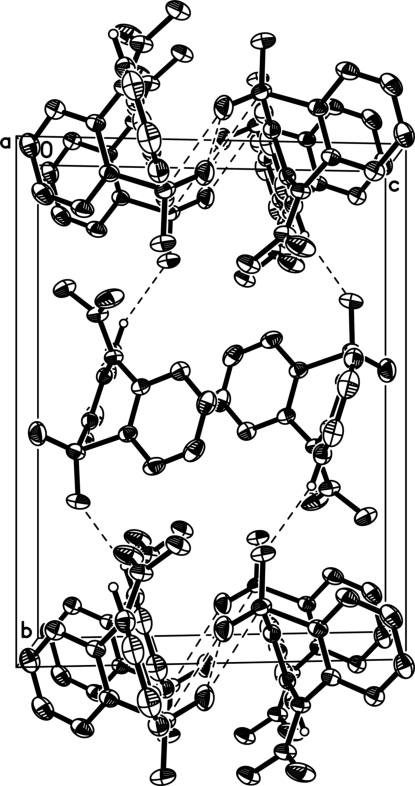
A partial packing diagram of the title compound. Hydrogen bonds are shown as dashed lines, viewed down the a axis.

## supplementary crystallographic information

### Comment

Phenothiazine is a well known heterocycle. The phenothiazine structure occurs in many synthetic dyes, electroluminescent materials (Miller *et al.*, 1999) and drugs, especially various antipsychotic drugs, *e.g*. Chlorpromazine and antihistaminic drugs, *e.g*. Promethazine (Wermuth, 2003). Recently, researchers find some new applications for phenothiazine derivatives in medicine, such as antitubercular (Wang *et al.*, 2008) and antitumor (Lam *et al.*, 2001). As a part of our program devoted to the new applications of phenothiazine derivatives in medicine, we report herein the crystal structure of the title compound, (I).

The molecular structure is shown in fig. 1, with the labeling scheme. The sulfoxide O atom is disordered over two sites (O1A and O1B) with occupancies of 0.88 and 0.12, respectively, corresponding to an inversion of the lone pair at tetrahedral S1 site.

The crystal structure of (I) consists of the self-assembly of the molecules through weak hydrogen bonding interactions of the kind C—H···O. The crystal packing (Fig. 2) consists of a wavy-like arrangement in the *ab* plane generated by intermolecular interactions of hydrogen bond between the O1A atom of sulfoxide and H atom H5 of the aromatic ring. On the other hand, π–π contacts between the aromatic rings [centroid to centroid distances = 3.7547 (15) and 3.9577 (15) Å ] in parallel accumulation may further stabilize the crystal structure.

### Experimental

All reagents were of analytical grade. The title sample was prepared according to a literature method (Gilman & Nelson, 1953; Chan *et al.*, 1998) from the *N*-benzylphenothiazine. The purity of the compound was checked by determining its melting point. It was characterized by recording its infrared spectra and elemental analyses. Single crystals of the title compound were obtained by slow evaporation of an ethanol solution. The X-ray diffraction studies were made at room temperature.

### Refinement

H atoms bonded to C atoms were positioned geometrically (C—H = 0.93 and 0.96Å for benzene and methyl H atoms, respectively) and included in the refinement in the riding-model approximation, with *U*_iso_(H) = 1.2 or 1.5 *U*_eq_(C). The sulfoxide O atom is disordered over two positions with partial site-occupancies of 0.88 and 0.12, respectively, which were fixed in the last least-squares cycles.

### Crystal data

**Table:**

C_14_H_11_NO_2_S	*F*(000) = 536
*M**_r_* = 257.30	*D*_x_ = 1.405 Mg m^−^^3^
Monoclinic, *P*2_1_/*n*	Melting point: 443 K
Hall symbol: -P 2yn	Mo *K*α radiation, λ = 0.71073 Å
*a* = 8.1244 (1) Å	Cell parameters from 4917 reflections
*b* = 14.1787 (2) Å	θ = 2.4–27.7°
*c* = 10.7576 (1) Å	µ = 0.26 mm^−^^1^
β = 100.963 (1)°	*T* = 296 K
*V* = 1216.59 (3) Å^3^	Block, orange
*Z* = 4	0.20 × 0.14 × 0.13 mm

### Data collection

**Table:**

Bruker APEXII CCD area-detector diffractometer	3067 independent reflections
Radiation source: fine-focus sealed tube	2404 reflections with *I* > 2σ(*I*)
graphite	*R*_int_ = 0.019
φ and ω scans	θ_max_ = 28.5°, θ_min_ = 2.4°
Absorption correction: multi-scan (*SADABS*; Sheldrick, 2003)	*h* = −10→10
*T*_min_ = 0.950, *T*_max_ = 0.967	*k* = −18→18
11538 measured reflections	*l* = −14→14

### Refinement

**Table:**

Refinement on *F*^2^	Primary atom site location: structure-invariant direct methods
Least-squares matrix: full	Secondary atom site location: difference Fourier map
*R*[*F*^2^ > 2σ(*F*^2^)] = 0.046	Hydrogen site location: inferred from neighbouring sites
*wR*(*F*^2^) = 0.127	H-atom parameters constrained
*S* = 1.09	*w* = 1/[σ^2^(*F*_o_^2^) + (0.0506*P*)^2^ + 0.5122*P*] where *P* = (*F*_o_^2^ + 2*F*_c_^2^)/3
3067 reflections	(Δ/σ)_max_ = 0.001
174 parameters	Δρ_max_ = 0.34 e Å^−^^3^
2 restraints	Δρ_min_ = −0.24 e Å^−^^3^
0 constraints	

### Fractional atomic coordinates and isotropic or equivalent isotropic displacement parameters (Å^2^)

**Table:**

	*x*	*y*	*z*	*U*_iso_*/*U*_eq_	Occ. (<1)
C1	0.2768 (2)	1.03984 (14)	0.34599 (17)	0.0419 (4)	
C2	0.4325 (3)	1.08344 (17)	0.3653 (2)	0.0552 (6)	
H2A	0.4440	1.1461	0.3911	0.066*	
C3	0.5687 (3)	1.0329 (2)	0.3456 (2)	0.0679 (7)	
H3A	0.6736	1.0614	0.3585	0.081*	
C4	0.5520 (3)	0.9400 (2)	0.3069 (2)	0.0603 (6)	
H4A	0.6461	0.9059	0.2960	0.072*	
C5	0.3958 (2)	0.89724 (16)	0.28427 (19)	0.0482 (5)	
H5A	0.3845	0.8353	0.2554	0.058*	
C6	0.2563 (2)	0.94696 (13)	0.30478 (16)	0.0372 (4)	
C7	−0.0305 (2)	0.97033 (13)	0.19798 (17)	0.0383 (4)	
C8	−0.0329 (2)	1.06443 (13)	0.23222 (18)	0.0415 (4)	
C9	−0.1414 (3)	1.12817 (16)	0.1596 (2)	0.0552 (5)	
H9A	−0.1414	1.1914	0.1824	0.066*	
C10	−0.2486 (3)	1.09580 (18)	0.0533 (2)	0.0600 (6)	
H10A	−0.3219	1.1375	0.0040	0.072*	
C11	−0.2480 (3)	1.00256 (18)	0.0197 (2)	0.0580 (6)	
H11A	−0.3223	0.9815	−0.0515	0.070*	
C12	−0.1381 (2)	0.93909 (15)	0.09038 (19)	0.0467 (5)	
H12A	−0.1369	0.8763	0.0657	0.056*	
C13	0.0405 (3)	0.82363 (14)	0.3156 (2)	0.0479 (5)	
C14	0.1638 (3)	0.76880 (16)	0.4099 (2)	0.0602 (6)	
H14A	0.1048	0.7321	0.4622	0.090*	
H14B	0.2386	0.8116	0.4619	0.090*	
H14C	0.2271	0.7276	0.3659	0.090*	
N1	0.09014 (18)	0.90973 (10)	0.27386 (15)	0.0388 (3)	
O2	−0.1007 (2)	0.79521 (12)	0.2791 (2)	0.0756 (6)	
S1	0.09806 (7)	1.10145 (4)	0.37655 (5)	0.05019 (17)	
O1A	0.1306 (3)	1.20324 (11)	0.37154 (19)	0.0716 (7)	0.886 (4)
O1B	0.0214 (17)	1.0640 (10)	0.4755 (10)	0.068 (5)	0.114 (4)

### Atomic displacement parameters (Å^2^)

**Table:**

	*U*^11^	*U*^22^	*U*^33^	*U*^12^	*U*^13^	*U*^23^
C1	0.0440 (10)	0.0426 (10)	0.0387 (9)	−0.0069 (8)	0.0070 (7)	0.0010 (8)
C2	0.0588 (13)	0.0576 (13)	0.0471 (11)	−0.0228 (11)	0.0047 (9)	0.0040 (9)
C3	0.0425 (11)	0.100 (2)	0.0595 (14)	−0.0217 (13)	0.0069 (10)	0.0140 (14)
C4	0.0384 (10)	0.0930 (19)	0.0505 (12)	0.0062 (11)	0.0108 (9)	0.0122 (12)
C5	0.0444 (10)	0.0558 (12)	0.0440 (10)	0.0094 (9)	0.0074 (8)	0.0036 (9)
C6	0.0354 (9)	0.0394 (10)	0.0362 (9)	−0.0009 (7)	0.0050 (7)	0.0030 (7)
C7	0.0326 (8)	0.0359 (9)	0.0469 (10)	0.0023 (7)	0.0090 (7)	0.0043 (7)
C8	0.0413 (10)	0.0369 (9)	0.0491 (10)	0.0044 (8)	0.0153 (8)	0.0038 (8)
C9	0.0607 (13)	0.0416 (11)	0.0674 (14)	0.0151 (10)	0.0230 (11)	0.0118 (10)
C10	0.0512 (12)	0.0671 (15)	0.0618 (13)	0.0193 (11)	0.0109 (10)	0.0222 (11)
C11	0.0445 (11)	0.0760 (16)	0.0510 (12)	0.0027 (11)	0.0028 (9)	0.0102 (11)
C12	0.0416 (10)	0.0466 (11)	0.0512 (11)	−0.0019 (9)	0.0072 (8)	0.0018 (9)
C13	0.0452 (11)	0.0334 (10)	0.0637 (13)	0.0001 (8)	0.0065 (9)	0.0048 (9)
C14	0.0633 (14)	0.0438 (12)	0.0713 (14)	0.0045 (10)	0.0071 (11)	0.0160 (10)
N1	0.0348 (7)	0.0305 (7)	0.0496 (9)	0.0005 (6)	0.0040 (6)	0.0027 (6)
O2	0.0546 (10)	0.0521 (10)	0.1130 (15)	−0.0162 (8)	−0.0019 (9)	0.0240 (9)
S1	0.0644 (3)	0.0362 (3)	0.0524 (3)	−0.0005 (2)	0.0175 (2)	−0.0066 (2)
O1A	0.1022 (16)	0.0319 (9)	0.0786 (14)	−0.0026 (9)	0.0119 (11)	−0.0088 (8)
O1B	0.084 (11)	0.080 (11)	0.045 (8)	0.017 (8)	0.020 (7)	−0.003 (7)

### Geometric parameters (Å, °)

**Table:**

C1—C2	1.387 (3)	C8—S1	1.786 (2)
C1—C6	1.389 (3)	C9—C10	1.377 (3)
C1—S1	1.778 (2)	C9—H9A	0.9300
C2—C3	1.369 (4)	C10—C11	1.371 (3)
C2—H2A	0.9300	C10—H10A	0.9300
C3—C4	1.380 (4)	C11—C12	1.388 (3)
C3—H3A	0.9300	C11—H11A	0.9300
C4—C5	1.385 (3)	C12—H12A	0.9300
C4—H4A	0.9300	C13—O2	1.209 (2)
C5—C6	1.388 (3)	C13—N1	1.387 (2)
C5—H5A	0.9300	C13—C14	1.500 (3)
C6—N1	1.428 (2)	C14—H14A	0.9600
C7—C12	1.384 (3)	C14—H14B	0.9600
C7—C8	1.385 (3)	C14—H14C	0.9600
C7—N1	1.436 (2)	S1—O1B	1.433 (5)
C8—C9	1.394 (3)	S1—O1A	1.4700 (17)
			
C2—C1—C6	121.40 (19)	C11—C10—C9	120.4 (2)
C2—C1—S1	120.45 (17)	C11—C10—H10A	119.8
C6—C1—S1	118.14 (14)	C9—C10—H10A	119.8
C3—C2—C1	119.0 (2)	C10—C11—C12	121.0 (2)
C3—C2—H2A	120.5	C10—C11—H11A	119.5
C1—C2—H2A	120.5	C12—C11—H11A	119.5
C2—C3—C4	120.7 (2)	C7—C12—C11	119.3 (2)
C2—C3—H3A	119.6	C7—C12—H12A	120.4
C4—C3—H3A	119.6	C11—C12—H12A	120.4
C3—C4—C5	120.3 (2)	O2—C13—N1	120.32 (18)
C3—C4—H4A	119.9	O2—C13—C14	121.16 (19)
C5—C4—H4A	119.9	N1—C13—C14	118.48 (18)
C4—C5—C6	119.9 (2)	C13—C14—H14A	109.5
C4—C5—H5A	120.0	C13—C14—H14B	109.5
C6—C5—H5A	120.0	H14A—C14—H14B	109.5
C5—C6—C1	118.67 (17)	C13—C14—H14C	109.5
C5—C6—N1	122.65 (17)	H14A—C14—H14C	109.5
C1—C6—N1	118.40 (16)	H14B—C14—H14C	109.5
C12—C7—C8	119.46 (17)	C13—N1—C6	124.75 (15)
C12—C7—N1	122.58 (17)	C13—N1—C7	120.12 (15)
C8—C7—N1	117.90 (16)	C6—N1—C7	115.08 (14)
C7—C8—C9	121.0 (2)	O1B—S1—O1A	119.8 (6)
C7—C8—S1	118.55 (14)	O1B—S1—C1	116.2 (6)
C9—C8—S1	120.37 (16)	O1A—S1—C1	108.47 (11)
C10—C9—C8	118.8 (2)	O1B—S1—C8	105.4 (6)
C10—C9—H9A	120.6	O1A—S1—C8	109.77 (10)
C8—C9—H9A	120.6	C1—S1—C8	93.87 (9)
			
C6—C1—C2—C3	−1.4 (3)	C14—C13—N1—C6	6.2 (3)
S1—C1—C2—C3	177.45 (17)	O2—C13—N1—C7	6.8 (3)
C1—C2—C3—C4	0.2 (3)	C14—C13—N1—C7	−171.17 (19)
C2—C3—C4—C5	1.7 (3)	C5—C6—N1—C13	54.0 (3)
C3—C4—C5—C6	−2.3 (3)	C1—C6—N1—C13	−132.1 (2)
C4—C5—C6—C1	1.1 (3)	C5—C6—N1—C7	−128.52 (19)
C4—C5—C6—N1	174.92 (18)	C1—C6—N1—C7	45.4 (2)
C2—C1—C6—C5	0.8 (3)	C12—C7—N1—C13	−51.1 (3)
S1—C1—C6—C5	−178.09 (14)	C8—C7—N1—C13	131.6 (2)
C2—C1—C6—N1	−173.37 (17)	C12—C7—N1—C6	131.29 (19)
S1—C1—C6—N1	7.8 (2)	C8—C7—N1—C6	−46.0 (2)
C12—C7—C8—C9	−0.4 (3)	C2—C1—S1—O1B	−116.3 (7)
N1—C7—C8—C9	177.03 (17)	C6—C1—S1—O1B	62.6 (7)
C12—C7—C8—S1	176.25 (15)	C2—C1—S1—O1A	22.1 (2)
N1—C7—C8—S1	−6.3 (2)	C6—C1—S1—O1A	−159.03 (15)
C7—C8—C9—C10	0.9 (3)	C2—C1—S1—C8	134.44 (17)
S1—C8—C9—C10	−175.71 (16)	C6—C1—S1—C8	−46.70 (16)
C8—C9—C10—C11	−0.2 (3)	C7—C8—S1—O1B	−72.4 (6)
C9—C10—C11—C12	−1.0 (3)	C9—C8—S1—O1B	104.2 (6)
C8—C7—C12—C11	−0.8 (3)	C7—C8—S1—O1A	157.27 (16)
N1—C7—C12—C11	−178.08 (18)	C9—C8—S1—O1A	−26.1 (2)
C10—C11—C12—C7	1.5 (3)	C7—C8—S1—C1	46.07 (16)
O2—C13—N1—C6	−175.9 (2)	C9—C8—S1—C1	−137.28 (17)

### Hydrogen-bond geometry (Å, °)

**Table:**

*D*—H···*A*	*D*—H	H···*A*	*D*···*A*	*D*—H···*A*
C5—H5A···O1A^i^	0.93	2.31	3.207 (3)	163

Symmetry codes: (i) −*x*+1/2, *y*−1/2, −*z*+1/2.
